# Role of *OCT1* and *MAP3K5* Genetic Polymorphisms in Hydroxyurea Pharmacokinetics

**DOI:** 10.3390/life15081284

**Published:** 2025-08-13

**Authors:** Sarah Allegra, Giuliana Abbadessa, Francesco Chiara, Daniela Di Grazia, Cristina Mirabella, Maura Caudana, Marina Zanatta, Jenni Bertello, Vincenzo Voi, Silvia De Francia

**Affiliations:** 1 Clinical Pharmacology, Department of Clinical and Biological Sciences, University of Turin, Orbassano, 10043 Turin, Italy; giuliana.abbadessa@unito.it (G.A.); francesco.chiara@unito.it (F.C.); daniela.digrazia@unito.it (D.D.G.); cristina.mirabella@edu.unito.it (C.M.); maura.caudana@unito.it (M.C.); silvia.defrancia@unito.it (S.D.F.); 2 Department of Physics, University of Trento, 38122 Povo, Italy; 3 Department of Economics and Statistics “Cognetti de Martiis”, University of Turin, 10124 Turin, Italy; marina.zanatta@unito.it; 4 Reference Centre for Haemoglobinopathies, Department of Clinical and Biological Sciences, University of Turin, 10124 Turin, Italy; jenni.bertello@edu.unito.it (J.B.); vincenzo.voi@unito.it (V.V.)

**Keywords:** hydroxycarbamide, pharmacogenetics, OCT1, MAP3K5

## Abstract

Background: Hydroxyurea is approved for the treatment of paediatric and adult sickle cell disease patients. It causes the synthesis of foetal haemoglobin and decreases platelets and granulocytes, but with a high interindividual variability, requiring higher dosages and escalating toxicity. Hereditary variables should be investigated to personalise treatment. We evaluated the possible influences of *OCT1* and *MAP3K5* gene polymorphisms on hydroxyurea pharmacokinetics. Methods: We conducted a retrospective analysis on 79 treated patients. The polymorphisms of *OCT1* (rs683369 G > C) and *MAP3K5* (rs9376230 C > A and rs9483947 C > T) were genotyped. Results: Sub-Saharan patients with the *OCT1* rs683369 GG genotype showed a lower drug half-life, compared to those with the GC genotype. In sub-Saharan paediatric female patients, the *OCT1* rs683369 GG genotype was associated with a lower t1/2 than the GC genotype. Conclusions: The findings demonstrate for the first time how crucial it is to assess the pharmacogenetics of hydroxyurea by taking into account the two sexes in different groups. Additionally, the data were evaluated with consideration for ethnic groups and individually for adults and children. Pharmacogenetic studies could improve the clinical management of hydroxyurea.

## 1. Introduction

Hydroxyurea (Droxia, Bristol-Myers-Squibb, New York, NY, USA), also called hydroxycarbamide, is a hydroxylated analogue of urea approved by the FDA in 1998 for the treatment of adult sickle cell disease (SCD) patients, and in 2017 it was approved for use in paediatric SCD patients older than two years. HU is still frequently used as the initial treatment for SCD [1]. It causes the synthesis of foetal haemoglobin (HbF) and decreases platelets and granulocytes, which lowers vaso-occlusive episodes and red blood cell sickling. HbF reactivation levels, however, differ from patient to patient and need to be maintained with lifelong therapy, requiring higher dosages and escalating toxicity [2,3,4]. Patients who do not need lifetime transfusion therapy to survive are referred to as having non-transfusion-dependent β-thalassemia (NTDT) [5]. βthalassemia intermedia, HbE/β-thalassemia, HbE illness, and α-thalassemia intermedia are among the clinically different variants of NTDT. Increased intestinal iron absorption and unsuitably low hepcidin levels are the results of inadequate erythropoiesis in NTDT. Ferroportin, intestinal iron absorption, and the release of recycled iron from the reticuloendothelial system all arise as a result of the ensuing low hepcidin levels. Systemic iron excess is caused by several processes. Because hepatocytes have more iron deposition than macrophages, NTDT patients have higher total body iron but lower serum ferritin than is typical [5].

Approximately 25% of patients are regarded as poor metabolizers or do not respond to HU treatment [6]. Finding the variants that might predict this response to HU is essential because HU is known to cause gastrointestinal toxicity, which includes nausea and anorexia, cytopenia, hyperpigmentation, weight gain, and possibly teratogenic effects. Using this knowledge, when therapeutic alternatives become available in the future, HU would not be prescribed to people who will not respond to treatment, saving time and money and preventing people from being exposed to a drug that could cause side effects [4,7,8]. Some of the most significant predictors of differences in drug therapy efficacy and tolerance are thought to be hereditary variables [9]. In addition to polymorphisms within the β-globin gene (HBB), recent research revealed that mutations in genes outside of HBB are also strongly linked to an increase in HbF levels and, as a result, HU treatment responsiveness [9]. For example, the effectiveness of HU therapy was linked to genetic variations of the MAP3K5, NOS2A, and ARG2 genes [10]. The FLT1 and ARG2 genetic variations were similarly linked to a mild NTDT patient phenotype, according to the same study. According to Kolliopoulou et al. [11], the MAP3K5, NOS2A, ARG2, and FLT1 genomic variants may be used as genomic biomarkers to describe the severity of the disease in patients with β-type hemoglobinopathies and to predict the effectiveness of HU therapy in Hb S-β-thalassemia compound heterozygotes. In addition, catalase, urease, horseradish peroxidase, and CYP450 family enzymes may be involved, according to experimental data from research that investigated the enzymatic metabolism of HU in order to better understand its mode of action [12,13].

As a member of the MAP kinase family, mitogen-activated protein kinase kinase 5 (MAP3K5), often referred to as apoptosis signal-regulating kinase 1 (ASK1), is a component of the mitogen-activated protein kinase pathway [14]. In response to a variety of stressors, including oxidative stress, endoplasmic reticulum stress, and calcium influx, it acts in a Raf-independent manner to activate c-Jun N-terminal kinase (JNK) and p38 mitogen-activated protein kinases [15]. MAP3K5 has been linked to cardiovascular and neurological conditions, rheumatoid arthritis, diabetes, and cancer. The *MAP3K5* gene is found at locus 6q22.33 on chromosome 6 and the transcribed protein has 11 kinase subdomains and 1374 amino acids; the human heart and pancreas contain large amounts of MAP3K5 transcript. In circumstances without stress, the MPA3K5 C-terminal coiled-coil domain (CCC) oligomerizes it, which is necessary for its activation. However, decreased thioredoxin (Trx), calcium and integrin binding protein 1 (CIB1) inhibit it, keeping it in an inactive state. Trx directly binds to the N-terminal coiled-coil domain (NCC) of MAP3K5 kinase, inhibiting its activity. Kinase activation is controlled by Trx and CIB1 in ways that are sensitive to redox or calcium. Both seem to compete with the MAP3K5 activator TNF-α receptor-associated factor 2 (TRAF2). MAP3K5 then recruits TRAF2 and TRAF6 to create a bigger molecular mass complex. Thus, the kinase establishes homo-oligomeric bonds with both the CCC and the NCC, resulting in its complete activation. By activating the NF-kb protein RelA, inflammatory cytokines including TNF-α and IL-1 can trigger the transcription of the *MAP3K5* gene. This gene expression is both transcriptionally and post-transcriptionally regulated [16,17]. The gene variants rs9483947 and rs9376230 have been already linked to HU treatment [18].

By affecting drug absorption, metabolism, distribution, or elimination, the solute carrier (SLC) gene superfamily encodes a range of membrane transport proteins that play important roles in the pharmacokinetic and pharmacodynamic characteristics of many medications [19,20]. The expression of renal and hepatic transporters probably has the biggest influence on drug pharmacology, despite the fact that SLC transporters are broadly dispersed [21]. Because they can transport xenobiotics in addition to ions, peptides, and proteins with varying sizes, shapes, and charges, SLC-family proteins like organic anion transporters, organic cation transporters (OCTs), organic cation/carnitine transporters and organic anion transporting polypeptides are drug transporters [22]. HU, like many other medications, diffuses passively through cell membranes. In contrast, before some urea transporters were discovered and kinetic investigations revealed enhanced diffusion pathways, it was previously believed that urea also used passive diffusion [21,23,24]. In vitro investigations showed that HU is a substrate for certain SLC transporters and that its passive diffusion transport across an artificial lipid bilayer is limited [25]. With a substitution of the amino acid phenylalanine to leucine (F160L) at position 160 in exon 2, *OCT1* rs683369 is one of the six most prevalent polymorphisms of the SLC22A1 gene [26]. Findings of a meta-analysis showed that, like the 1222 G > A mutation, rs683369 may impact OCT-1 protein activity by decreasing drugs’ therapeutic efficacy [27,28].

In the present pharmacogenomic study, we evaluated the possible influences of the OCT1 and MAP3K5 polymorphisms on HU pharmacokinetics. The data were analysed, taking into account differences in sex, age and ethnicity.

## 2. Materials and Methods

We conducted a retrospective analysis on 79 SCD patients who were enrolled between July 2023 and March 2024 and received treatment at the Hemoglobinopathies University Centre of San Luigi Gonzaga Hospital in Orbassano (Turin, Italy). The local Ethics Committee approved the study protocol for the study, entitled “Exploration study of pharmacokinetics and pharmacogenetics of the drug hydroxyurea in patients with beta-hemoglobinopathy: non-transfusion dependent beta thalassemia and sickle cell anemia,” abbreviated to PKPG-Betahb. Every enrolled subject gave their written informed consent for the study. To guarantee a broad population, the study included patients of all ages and backgrounds and was conducted in accordance with the patients’ availability and regular follow-up visit schedule. Patients who were either naïve or had previously had treatment with HU (Siklos^®^ tablets) were enrolled in the research, following a 48 h washout period. The washout time was defined as the time required for the drug and its effects to completely disappear from the body. Given the short half-life of HU, our team defined 48 h as sufficient time to achieve this goal. The following information was accessible and gathered for each patient: sex, age, HU dose, therapy start date, BMI, BSA, and ethnicity. Unfortunately, data regarding the SCD genotype were not collected. Patients who started concomitant therapies during the study were excluded.

Samples taken prior to drug consumption as well as 2, 4, 6, and 24 h following drug delivery were used to measure plasma HU concentrations; this will provide sufficient data to define the most important pharmacokinetic parameters in the study population. Within 30 min of collection, blood samples (collected in a 5 mL lithium–heparin tube) were centrifuged at 50 rounds/s (Hz) for 10 min at 4 °C, and plasma was kept in cryovials at −20 °C prior to analysis. HU concentrations were measured using an HPLC-UV technique [29]. The pharmacokinetic parameters, such as maximum concentration (Cmax), area under the curve (AUC), elimination half-life (t1/2), volume of distribution (Vd), minimum concentration (Cmin) and time to reach the maximum concentration (Tmax) were calculated using non-compartmental analysis.

A QIamp DNA Mini Kit (Quiagen, Valencia, CA, USA) was used to extract the DNA. The polymorphisms of OCT1 (rs683369 G > C) and MAP3K5 (rs9376230 C > A and rs9483947 C > T) were genotyped utilizing TaqMan assays (Applied Biosystems, Foster City, CA, USA). The Applied Biosystems ABI 7900HT Fast Real-Time PCR System was used for both amplification and dissociation. The negative derivative of the fluorescence change was computed automatically by the PCR system. The quality controls used were:

- Negative control: To ensure that the fluorescent signal is not the result of contamination or background noise, a negative control sample is one that does not contain the target DNA.
- Positive controls: A sample with a known genotype that is used to confirm the assay functionality and guarantee reliable results.
- Duplicate samples: To evaluate the overall experimental process variability, encompassing both sampling and analysis.

In order to assess the statistical dispersion of the data, continuous and non-normal variables were presented as median and the interquartile range (IQR, quartile 1; quartile 3). Frequency and percentage were used to describe categorical variables. The Shapiro–Wilk test was used to determine whether each variable was normal. The Kolmogorov–Smirnov test was used to assess each parameter’s correlation with a normal or non-normal distribution.

The following formula, where p2 = frequency of homozygous wild genotype, 2pq = frequency of heterozygous genotype, and q2 = frequency of homozygous mutant genotype, was used to evaluate Hardy–Weinberg equilibrium: p2 + 2pq + q2 = 1. The observed and expected genotype frequencies were compared using the Chi-squared test. The linkage disequilibrium was calculated using the https://ldlink.nih.gov/ tool (accessed on 30 May 2025). The difference between the actual and expected haplotype frequencies, assuming linkage equilibrium, is denoted as D’. The squared correlation between the genotypes at the two loci is denoted by R^2^.

The impact of SNPs on pharmacokinetic parameters was assessed using the Kruskal–Wallis and Mann–Whitney tests, taking into account the level of statistical significance (*p* value < 0.05). Finally, univariate and multivariate linear regression analyses were used to assess the predictive potential of the variables under consideration. Multivariate analysis (*p* value < 0.05) included factors (β, β coefficient; IC, interval of confidence at 95%) with a *p* value < 0.2 in univariate analysis. IBM SPSS Statistics 25.0 (Chicago, IL, USA) was used for all of the tests.

## 3. Results

### 3.1. Study Population

Seventy-nine patients were enrolled. Table 1 summarises the demographic, clinical, and pharmacokinetic characteristics of our cohort.

### 3.2. Hardy–Weinberg and Linkage

**Figure 1:**
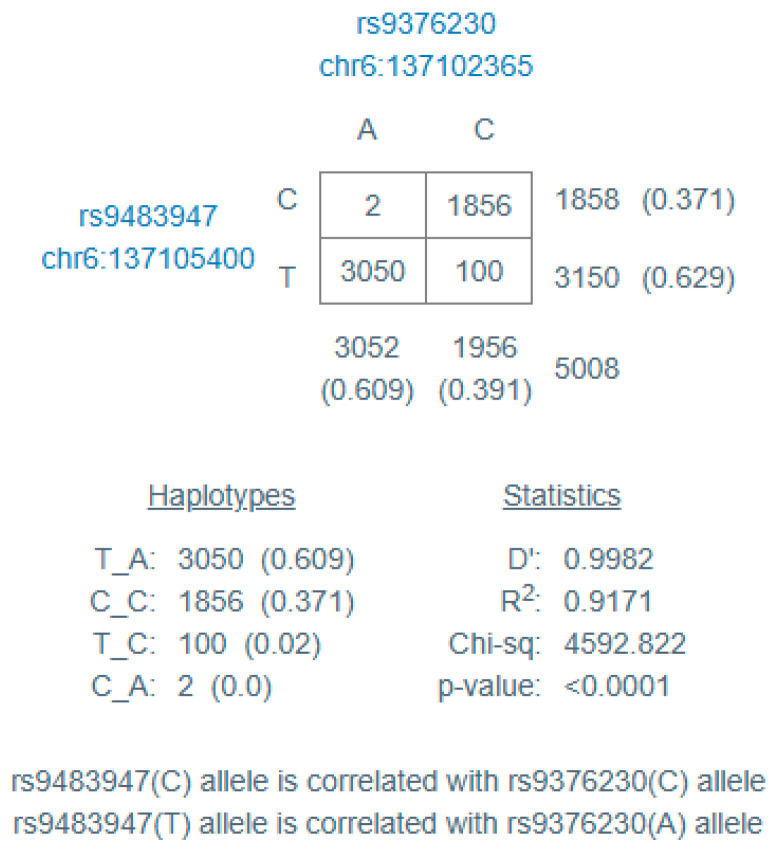
Linkage disequilibrium results, calculated with https://ldlink.nih.gov/ (accessed on 10 May 2025). D’ = difference between the actual and expected haplotype frequencies, assuming linkage equilibrium; R^2^ = squared correlation between the genotypes at the two loci.

The two variants evaluated in the MAP3K5 gene were in linkage disequilibrium, as described in Figure 1.

### 3.3. Effect of Pharmacogenetic on HU Pharmacokinetics

**Figure 2:**
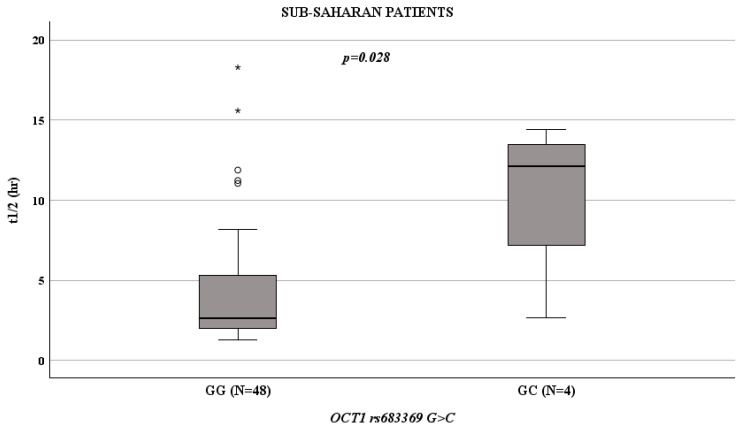
Boxplots showing the associations between t1/2 and *OCT1* rs683369 genotype in Sub-Saharan patients. Boxes and black lines in boxes represent respectively interquartile ranges (IQR) and median values; open dots and stars represent outlier values. Median values (horizontal line), IQR (bars), patient values (black square), highest and lowest value (whiskers) and *p* value are shown. OCT1 rs683369 GG (N = 48; median t1/2 value 2.61 h; IQR: 2.01–5.43 h) versus GC (N = 4; median t1/2 value: 12.1 h; IQR: 4.94–13.93 h).

Given the importance and strong weight of ethnicity in genetic evaluations, we decided to evaluate the ethnicities of our cohort separately. In Sub-Saharan patients, *OCT1* rs683369 GG influenced t1/2 values (*p* = 0.028; Figure 2): compared to patients with a GC genotype (median value: 12.1 h; IQR: 4.94–13.93 h), those with a GG genotype had a reduced t1/2 (median value: 2.61 h; IQR: 2.01–5.43 h).

Evaluating adult patients and paediatric patients in two separate groups, Mann–Whitney tests revealed that no genetic factors were associated with liver HU pharmacokinetics. However, considering only the group of Sub-Saharan paediatric female patients, *OCT1* rs683369 GG influenced t1/2 (*p* = 0.047): patients with a GG genotype showed lower t1/2 (median value: 2.41 h; IQR: 1.82–5 h) than those with a GC genotype (median value: 13.08 h; IQR: 11.75–14.41 h). In the population under investigation, the *OCT1* rs683369 GC genotype was absent.

## 4. Discussion

Over 7 million people worldwide are diagnosed with SCD, the most common genetic monogenic blood illness. A point mutation in the adult β-globin gene causes SCD by producing aberrant haemoglobin S, which polymerises in the presence of dioxygen [30]. Red blood cell (RBC) sickling, acute painful vaso-occlusive episodes, persistent haemolytic anaemia, and progressive tissue and organ destruction are the overall outcomes [31]. Every year, around 400,000 babies are born with sickle cell disease (SCA), and 79% of the 7.7 million sickle cell disease patients reside in sub-Saharan Africa [32]. HU has been used to treat a large number of SCD patients to date, and those who have reacted favourably have seen a significant improvement in their quality of life. It has been suggested that inadequate medication dosage and treatment compliance are important factors that influence the HU response [33]. Because of their impact on drug metabolism, genetic variables are expected to have a role in this phenomenon [25,34,35]. However, the majority of HU pharmacogenomics research has been carried out in patients of non-sub-Saharan ethnicities [11,12,34,36,37,38,39,40,41].

Here we reported a preliminary study on HU pharmacogenetics in a mixed cohort of Caucasian, sub-Saharan, adult and paediatric SCD patients treated with HU.

First, we observed that the results differ based on ethnicity and age, suggesting that these factors should be taken into account when evaluating HU treatment, and unlike what has previously been reported [42]. African American and African patients make up a significant percentage of HU users; this might be because sickle cell disease is more common in these groups [43,44]. Although HU is useful in reducing sickle cell disease complications, research indicates that teenagers of all ethnic backgrounds may have trouble adhering to their treatment plans. Ethnicity is not the main determinant, but HU use and adherence can be influenced by age, socioeconomic level, and access to healthcare [42,43,44,45]. Recently, the HU PK profiles of 425 children—122 American, 262 African, and 42 Caribbean—were gathered and examined: there was regional variance in the patients’ HU dose (13–44 mg/kg), weight (7–88 kg), and age (range 0.5–18 years). However, clearance, volume of distribution and absorption did not differ. Globally, there was no significant difference in the dose required to attain the maximum tolerated dose and the results indicated no statistically significant differences in exposure [46]. On the other hand, other studies have reported that, regardless of the formulation, the PK profiles of oral HU are clearly defined and exhibit exceptional consistency in Cmax and AUC values across studies and age ranges [47,48,49,50,51]. In addition, HU PK results in newborns were comparable to those in older children and adults, with a large portion of the interpatient variability in the population being explained by body weight alone. As a result, weight-based dosing produces comparable systemic exposures, even for infants younger than two years, and age-related dosage modifications are not required [52].

Treatment response varies between men and women, mostly due to physiological, anatomical, and hormonal factors. The response to therapies is influenced by the existence of variations in the pharmacokinetics and pharmacodynamics of therapeutic agents. Even though this has been known since 1932, when the first study on the gender difference in the pharmacology of barbiturates in rats was published, it was not until the end of the 20th century that the significance of gender pharmacology was fully recognised [53,54,55,56,57]. The absorption, distribution, metabolism, and elimination stages exhibit notable sex-related differences and are mostly impacted by age and hormones. Numerous pharmacodynamic variations exist based on sex, mostly influenced by hormones, DNA, and environmental factors. However, it is more difficult to identify pharmacodynamic differences than it is to study pharmacokinetic differences [53]. All organ systems undergo structural and functional changes as people age, which lowers their ability to maintain homeostasis. An increased susceptibility to stress is caused by the depletion of functional reserve, even when a system function may be preserved while at rest. A decrease in the clearance of water-soluble and lipid-soluble medications, and an increase in the volume of distribution of lipid-soluble pharmaceuticals are caused by changes in hepatic, renal, and body composition. The half-life of plasma elimination is prolonged as a result of all these modifications. There are also notable pharmacodynamic alterations that generally lead to heightened drug sensitivity [58]. Our previous study reported that male median levels (117.9 mg/L/h; IQR 73.6–355.9) were higher than female median levels (97.4 mg/L/h; IQR 71.9–336.8) [29].

Considering the pharmacogenomic results, we observed that, in Sub-Saharan patients, *OCT1* rs683369 GG was related to a lower HU half-life. By affecting drugs’ absorption, metabolism, distribution, or elimination, the solute carrier (SLC) gene superfamily encodes a range of membrane transport proteins that play important roles in the pharmacokinetic and pharmacodynamic characteristics of many medications [19,20]. The expression of renal and hepatic transporters has the biggest influence on drug pharmacology, even though SLC transporters are broadly dispersed [21]. Because they can transport xenobiotics in addition to ions, peptides, and proteins with varying sizes, shapes, and charges, SLC-family proteins like organic anion transporters (OATs), organic cation transporters (OCTs), organic cation/carnitine transporters (OCTNs), and organic anion transporting polypeptides (OATPs) are considered drug transporters [22]. The SLC superfamily also includes urea transporters (UT), which are proteins that move urea across cell membranes and are essential for kidney function, urine concentration, and urea recycling [59]. According to a few studies, urea analogues, such as hydroxyurea, may serve as UT substrates [60,61]. HU has been identified as a substrate for particular SLC drug transporters, such as the urea transporters, by in vitro uptake and transport studies [25]. It has also been reported that OCT1 is involved in HU transport [24]. The *SLC22A1* gene, which is found on chromosome 6q26 and has 11 exons totalling around 37 kb, encodes human OCT1 [62,63]. The hepatic absorption, distribution, and excretion of clinically significant medications are influenced by OCT1, which is mostly expressed in the sinusoidal or basolateral membrane of hepatocytes [64]. Prior research has demonstrated that ethnically varied groups exhibit significant levels of *OCT1* polymorphism [65,66]. According to earlier research, pharmacogenetically significant genes in South African populations display distinct allele frequencies and new genetic variation [67]. South Africa is home to numerous indigenous and immigrant population groups and there may be a considerable amount of genetic variability in the native populations [68]. The abundance of information and knowledge that this genomic variety may offer may one day be used to help us better understand how genetic variation affects inter-individual variability in treatment responses [69]. Genetic variations in the *OCT1* gene can lead to differences in how well OCT1 functions, which can affect how individuals respond to drugs that are OCT1 substrates. Sadhasivam et al. suggested a more common loss of OCT1 activity in Caucasians compared with Africans [70]. Other analyses showed that, in Sub-Saharan Africa, only 5% of the population carry alleles causing a reduction in OCT1-mediated uptake of morphine, in contrast to 25% in Europe [71]. The highly variable frequency of loss-of-function OCT1 polymorphisms suggests the existence of selection pressure for losing (or maintaining) OCT1 activity in some world regions [72]. Here, we observed an absence of the C OCT1 rs683369 allele in the sub-Saharan population, suggesting that a high percentage of these people have a shortened drug half-life: the drug acts quickly but loses its effects quickly, requiring many doses throughout the day to obtain the same effect.

It is important to draw attention to our study’s limitations. In order to properly highlight the pharmacology of HU, the paper is deficient in information about SCD genotype, HbF levels, nutritional status, renal/hepatic function, concomitant medications, clinical outcomes and adverse pharmacological events. In addition, the sample size is small and evenly dispersed. Furthermore, because this study is monocentric, additional research is required to validate our findings across several cohorts. In future, analysis of additional gene variations should be carried out, and the data obtained from the few cases with the GC genotype of *OCT1* rs683369 certainly need to be confirmed in larger populations.

At several levels, including genetic, the HU treatment response can be tracked or anticipated. Genetic markers can be identified before therapy begins, making them the greatest markers for predicting medication response, whereas molecular and haematological profiles can only be assessed after treatment. To summarise, pharmacogenetics has a lot of potential for enhancing medication therapy and patient results. A more individualised and successful approach to medication therapy is being made possible by continued research and initiatives to incorporate pharmacogenetic information into clinical practice, even though there are still obstacles in the way of its clinical adoption. The pharmacogenetics of HU, when they become accessible, should a priori predict the HU response, improving the choice between dose escalation and the need for different medications or therapies.

There is little data on pharmacokinetics- and pharmacodynamics-based HU therapy customization. Numerous studies currently in existence examine the relationships between genotypes and toxicity or responsiveness, but they offer scant information on the effectiveness of treatment individualization. The findings imply that there are notable variations in pharmacokinetic parameters between patients who respond and those who do not. Combining the two methods could improve pharmacotherapy because pharmacogenetic information may be helpful at the start of treatment. However, clinical trials are necessary in order to more precisely demonstrate their advantages.

## Figures and Tables

**Table 1:** Demographic, clinical and pharmacokinetic characteristics of the enrolled patients.

	ALL	PAEDIATRICS	ADULTS
Variable	N = 79	N = 54	N = 25
**Gender**			
Male, N (%)	37 (46.8)	20 (37)	17 (68)
Female, N (%)	42 (53.2)	34 (63)	8 (32)
**Age (years)**			
Median (IQR)	15.00 (8.1–21.3)	11.6 (6.65–14.8)	24.6 (20.78–48.15)
**Ethnicity**			
Caucasian, N (%)	18 (22.8)	4 (7.4)	14 (58.3)
Sub-Saharan, N (%)	61 (77.2)	50 (92.6)	11 (41.7)
**HU dose mg/day**			
Median (IQR)	750 (500–1000)	750 (500–1000)	750 (500–1000)
**HU AUC (mg/L/h)**			
Median (IQR)	97.36 (67.86–148.17)	77.33 (61.46–140.56)	120.92 (93.58–179.78)
**HU t1/2 (h)**			
Median (IQR)	2.69 (2.01–6.01)	2.41 (1.92–5.29)	5.08 (2.34–6.8)
**HU Cmax (mg/L)**			
Median (IQR)	16.35 (11.25–25.24)	14.59 (10.31–22.78)	19.64 (15.03–27.81)
**HU Tmax (h)**			
Median (IQR)	2 (2–2)	2 (2–2)	2 (2–2)

N = number, IQR = interquartile range (25th and 75th percentile), AUC = area under the concentration curve, t1/2 = half-life, Cmax = maximum concentration, Tmax = time to reach the maximum concentration.

## Data Availability

The original contributions presented in the study are included in the article; further inquiries can be directed to the corresponding author.
